# 

**Published:** 1886-03

**Authors:** 


### THE BRISTOL

### Medico-Chirurgical Journal.

A JOURNAL OF THE MEDICAL SCIENCES FOR THE WEST OF ENGLAND AND SOUTH WALES.

*PUBLISHED UNDER THE AUSPICES OF*

*THE BRISTOL MEDICO-CHIRURGICAL SOCIETY.*

EDITOR:

J. GREIG SMITH,

M.A., F.R.S.E.

ASSISTANT-EDITOR:

L. M. GRIFFITHS.

---

"Scire est nescire, nisi id me

Scire alius sciret."

---

SURGEON GEN'L'S OFFICE
LIBRARY
106751

*VOL. IV.*

BRISTOL: J. W. ARROWSMITH;
LONDON: J. & A. CHURCHILL, 11 NEW BURLINGTON STREET.
1886.

WI
BR323

J.W. ARROWSMITH,

Printer and

Publisher,

QUAY STREET, BRISTOL.

3375 [illegible]

### CONTENTS OF VOL. IV.

ORIGINAL PAPERS:— *Page.*

Removal of the Uterine Appendages. By J. GREIG SMITH ... 1, 73

Relation between Facial Neuralgia and Dental Irritation. By J. M. ACKLAND ... ... ... ... ... ... ... ... ... ... 28

The Laryngoscope in Medicine. By BARCLAY J. BARON, M.B. 145

Heart-Lesions in Relation to Mental Symptoms. By P. WM. MACDONALD, M.D. ... ... ... ... ... ... ... ... ... 162

The Recuperative Power of Old Age. By H. BOYLE RUNNALLS 168

Presidential Address. By F. POOLE LANSDOWN ... ... ... ... 217

The Treatment of Empyema by Incision and Drainage. By W. H. C. NEWNHAM, M.B. ... ... ... ... ... ... ... 225

CLINICAL RECORDS:—

A Case of Dislocation of the Ulna Backwards and the Radius Forwards. By R. J. H. SCOTT ... ... ... ... ... ... 36

Calculus on Foreign Body in Bladder; Perforation of Bladder. No Symptoms. By H. BENHAM, M.D., and J. GREIG SMITH 38

Tonic Contraction of the Lower Extremities, or Tetany. By W. H. WEBB ... ... ... ... ... ... ... ... ... ... 42

The Vomiting of Pregnancy. By JOHN MEREDITH, M.D. ... 95

Some Unusual Symptoms in a Case of Hydrocephalus. By W. BARRETT ROUÉ, M.D., M.S. ... ... ... ... ... ... ... 102

Case of Typhlitis. By J. G. SWAYNE, M.D. ... ... ... ... ... 108

Notes on a Case of Bronchial Croup. By G. ARTHUR BROWN ... 115

A Case of Masked Epilepsy. By BONVILLE B. FOX, M.A., M.D. ... ... ... ... ... ... ... ... ... ... ... ... 172

Page.

A Case of Intestinal Intussusception. By CHRISTOPHER ELLIOTT, M.D. ... ... ... ... ... ... ... ... ... ... ... ... 177

A Case of Emphysematous Abscess of Thigh, from Intestinal Injury. By G. HERBERT LILLEY, M.D. ... ... ... ... 181

Cases of Vesical Calculi. By CHARLES F. PICKERING ... ... 183

Some Remarks on the Surgery of the Upper Eyelid. By F. RICHARDSON CROSS, M.B. ... ... ... ... ... ... ... 186

Case of Pernicious Anæmia. By T. COLE, M.D. ... ... ... 231

Case of Aneurism of Aorta—Treatment by Galvano-Punctures. By R. SHINGLETON SMITH, M.D. ... ... ... ... ... ... 233

The Use of the Aspirator in Retention of Urine. By W. FAIRBANKS, M.D. ... ... ... ... ... ... ... ... ... ... ... ... 242

PERISCOPE OF MEDICAL AND SURGICAL PROGRESS ... 45, 119, 192, 248

NOTES ON PREPARATIONS FOR THE SICK ... ... ... ... ... ... 268

REVIEWS AND NOTICES OF BOOKS ... ... ... ... ... 60, 137, 206, 273

SCRAPS ... ... ... ... ... ... ... ... ... ... ... 71, 143, 215, 287

### Past Presidents

OF THE

### Bristol Medico-Chirurgical Society.

1874—75. FREDERICK BRITTAN, M.D., B.A. Dub.

1875—76. ROBERT WILLIAM COE.

1876—77. SAMUEL MARTYN, M.D. Ed.

1877—78. AUGUSTIN PRICHARD, M.D. Berlin.

1878—79. HENRY EDWARD FRIPP, M.D. Würzburg.

1879—80. WILLIAM MICHELL CLARKE.

1880—81. JOSEPH GRIFFITHS SWAYNE, M.D. Lond.

1881—82. EDWARD LONG FOX, M.D. Oxon.

1882—83. JAMES GEORGE DAVEY, M.D. St. And.

1883—84. WILLIAM JOHNSTONE FYFFE, M.D. Dub.

1884—85. GEORGE FORSTER BURDER, M.D. Aberd.

### 1886.

### LIST OF SUBSCRIBERS

TO THE

### Bristol Medico-Chirurgical Journal.

*Those marked * are Members of the Society.*

| *Name.* | *Residence.* |
|---|---|
| Ackland, J. Mackno | 24 Southernhay, Exeter. |
| Ackland, W. H., M.D. St. And. | Bideford. |
| Adams, George | 5 Oakfield Road, Clifton. |
| *Adams, John Barfield | 3 Selwood Villas, Bristol. |
| Alexander, James, M.D., M.Ch. Dub. | Bishop's Place, Paignton. |
| Alford, George Ernest | 5 Royal Crescent, Weston-super-Mare. |
| Alford, Henry J., M.D. Lond. | 20 The Crescent, Taunton. |
| *Andrews, Onslow, M.D. St. And. | 159 White Ladies Road, Bristol. |
| Anstie, Thomas B. | 21 Northgate Street, Devizes. |
| *Atchley George F., M.B. Lond. | Tyndall's Park, Bristol. |
| Baker, A. de W. | 2 Lawn Terrace, Dawlish. |
| Baretti, T. G. L'Enardi | 49 Royal York Crescent, Clifton. |
| *Baron, B. J., M.B., C.M. Ed. | 12 Richmond Hill, Clifton. |
| Basset, W. | Carlisle House, Totterdown, Bristol. |
| Batten, Rayner W., M.D. Lond. | 1 Brunswick Square, Gloucester |
| Batten, W. Smith | Curzon House, Calne. |
| Beatson, W. B., M.D. St. And. | 11 Cavendish Place, Bath. |
| *Beddoe, John, M.D. Ed., B.A. Lond., F.R.S. | 2 Lansdown Place, Clifton. |
| Benham, Harry A., M.D., C.M. Aberd. | City and County Asylum, Stapleton. |
| Bennett, W. S. | Escot, Penzance. |
| Berry, F. C., M.D., B.Ch. Dub. | Lynton. |
| Bisdée, Alfred J. | Eversleigh, Banwell. |
| *Blacker, A. E. | 7 Unity Street, Bristol. |
| Blacker, E. | Midsomer Norton. |
| *Blagg, Arthur F. | 6 West Mall, Clifton. |
| Blaxall, F. H., M.D. St. And. | Clan Lodge, Bath. |
| *Board, Edmund C. | 7 Caledonia Place, Clifton. |
| Bond, Francis T., M.D. Lond. | 1 Beaufort Buildings, Spa, Gloucester. |
| Bower, Ernest D. | 1 Norfolk Terrace, Gloucester. |
| Boyle, Thomas | New Quay, Cornwall. |
| *Boys, Arthur Henry | Lodway Villa, Pill. |
| Bramwell, J. W., M.D. Ed. | 6 Bayshill Villas, Cheltenham. |
| Bright, J. A. | Glastonbury. |
| Broom, John, M.D. Brussels | St. Paul's Road, Clifton. |
| Brown, G. Arthur | Tredegar. |
| Browne, A. E. Newbury | Burbage, Wiltshire. |
| Browne, G. Henry | The Hermitage, Brynmawr. |
| Browne, J. Walton, M.D., B.A. Qu. Univ. I. | 10 College Square North, Belfast. |
| Bryden, Richard | Uffculme. |
| Bubb, John | 6 Royal Crescent, Cheltenham. |
| *Burder, George F., M.D. Aberd. | 63 Oakfield Road, Clifton. |

### LIST OF SUBSCRIBERS.

| | |
|---|---|
| Burges, R. E., M.D., M.Ch., B.A. Qu. Univ. I. | Kettering. |
| Burroughs, E. F. H. ... ... ... ... ... ... | Rook Lane House, Frome. |
| *Bush, J. Paul ... ... ... ... ... ... ... ... | 2 Rodney Cottages, Clifton. |
| Cann, Francis M. ... ... ... ... ... ... ... ... | Sefton House, Dawlish. |
| Carey, J., M.D. Lond. ... ... ... ... ... ... | 2 Hamdon Villas, Taunton. |
| Carlyon, Thomas B. ... ... ... ... ... ... | Redruth. |
| *Carr, Alexander ... ... ... ... ... ... ... | Wells Road, Bristol. |
| Carter, R. W., M.D. Calcutta ... ... ... ... | 1 Bank Buildings, Weymouth. |
| *Carter, W. F.... ... ... ... ... ... ... ... | Coronation Road, Bristol. |
| Cave, Edward J. ... ... ... ... ... ... ... | Royal United Hospital, Bath. |
| *Challacombe, J. P., M.D. Ed. ... ... ... | 143 Cheltenham Road, Bristol. |
| Clark, Thomas ... ... ... ... ... ... ... | Blair Lodge, Minehead. |
| Clarke, Arthur, B.A. Lond. ... ... ... ... | Street, Somersetshire. |
| Clarke, Fred. H. ... ... ... ... ... ... ... ... | Chillington. |
| *Clarke, John Michell, M.B., B.A. Cantab. | 2 York Buildings, Clifton. |
| Clay, Challoner ... ... ... ... ... ... ... | Manor House, Fovant. |
| Clay, R. Hogarth, M.D. Ed. ... ... ... ... | 4 Windsor Villas, Plymouth. |
| Coates, Harcourt ... ... ... ... ... ... ... | 17 Endless Street, Salisbury. |
| Coathupe, Edwin W. ... ... ... ... ... ... | 7 Clifton Park, Clifton. |
| *Coe, R. W. ... ... ... ... ... ... ... ... ... | 14 Berkeley Square, Bristol. |
| Coker, T. V. ... ... ... ... ... ... ... ... ... | 2 Chesterfield Place, Clifton. |
| Cole, Thomas, M.D. Lond. ... ... ... ... | 18 Circus, Bath. |
| *Collins, H. W. ... ... ... ... ... ... ... ... | Wrington. |
| Collyns, Robert J. ... ... ... ... ... ... | Dulverton. |
| Colman, Thomas J., M.D. Ed.... ... ... ... | Redland Road, Bristol. |
| Colmer, Ptolemy S. H., M.D. Dur. ... ... | South Street House, Yeovil. |
| Colthurst, J. B. ... ... ... ... ... ... ... ... | Wincanton. |
| Cooke, A. S. ... ... ... ... ... ... ... ... ... | Badbrook House, Stroud. |
| Cooper, Herbert ... ... ... ... ... ... ... | Hampstead, London, N.W. |
| *Corbould, George G. ... ... ... ... ... | 22 Berkeley Square, Bristol. |
| Cornwall, James ... ... ... ... ... ... ... | Fairford. |
| Cowan, F. S. ... ... ... ... ... ... ... ... ... | 27 Queen Square, Bath. |
| Cox, William I. ... ... ... ... ... ... ... ... | Hawkesbury-Upton. |
| Crespi, Alfred J. H. ... ... ... ... ... | St. John's Hill, Wimborne. |
| Cripps, Edward Charles ... ... ... ... ... | Cirencester. |
| Crisp, James Henry ... ... ... ... ... ... | The Grange, Lacock. |
| *Crisp, Nathaniel ... ... ... ... ... ... ... | Chandos House, Keynsham. |
| *Cross, F. Richardson, M.B. Lond. ... ... | Chandos Villa, Clifton. |
| *Crossman, Edward ... ... ... ... ... ... | Hambrook. |
| Cunningham, A. G., M.B., C.M. Aberd. ... | Stapleton Road, Bristol. |
| Currie, Andrew S., M.D. Ed.... ... ... ... | Oakfield Villa, Lydney. |
| *Dacre, John ... ... ... ... ... ... ... ... | Royal Infirmary, Bristol. |
| *Daly, George H., M.D. St. And. ... ... ... | Burley House, Chippenham. |
| Daniel, T. P. ... ... ... ... ... ... ... ... ... | Beaminster. |
| *Davey, James G., M.D. St. And. ... ... ... | 4 Redland Park, Bristol. |
| *Davies, D. S., M.B. Lond. ... ... ... ... | 60 Oakfield Road, Clifton. |
| Davies, E. Cluneglas ... ... ... ... ... | Pontfaen, Lampeter. |
| Davies, John W. ... ... ... ... ... ... ... | Hillside House, Ebbw Vale. |
| Davis, A. A. ... ... ... ... ... ... ... ... | Fowey. |
| Davis, Theodore, M.D. Lond. ... ... ... | Beachcroft, Clevedon. |
| Day, Percy Howard ... ... ... ... ... ... | Stalmine Lodge, Poulton-le-Fylde. |
| Deas, P. Maury, M.B., M.S. Lond. ... ... | Wonford House, Exeter. |
| Dening, Edwin ... ... ... ... ... ... ... | Stow-on-the-Wold. |
| Devonald, Alfred ... ... ... ... ... ... | Llangennech. |

### LIST OF SUBSCRIBERS.

| | |
|---|---|
| *Dew, Henry R. ... ... ... ... ... ... ... | 25 Berkeley Square, Bristol. |
| *Dobson, Nelson C. ... ... ... ... ... ... ... | 27 Victoria Square, Clifton. |
| *Dowson, C. H. ... ... ... ... ... ... ... | Tyndall's Park, Bristol. |
| *Dowson, W., M.B., M.A. Cantab. ... ... ... | Great George Street, Bristol. |
| Doyne, R. W. ... ... ... ... ... ... ... ... | Southbourne, Oxford. |
| Dunbar, Eliza L. Walker, M.D. Zürich ... | 9 Oakfield Road, Clifton. |
| Duncan, William ... ... ... ... ... ... ... | Ridgeway, Frome. |
| Eadon, W. F. Bailey ... ... ... ... ... ... | Hambrook. |
| Eager, Reginald, M.D. Lond. ... ... ... | Northwoods, Winterbourne. |
| Eddowes, Charles ... ... ... ... ... ... | Maddington, Devizes. |
| Edgeworth, T. F.... ... ... ... ... ... ... | 1 Stokes Croft, Bristol. |
| Edwardes, Thomas ... ... ... ... ... ... | Glascoed, Llansaintffraid. |
| *Elliott, Christopher, M.D., B.A. Dub. ... | 88 Pembroke Road, Clifton. |
| Ellis, Henry V., M.B., C.M. Aberd. ... ... | Ty Bryn, Reynoldstone. |
| *Elwin, J. Fountain ... ... ... ... ... ... | 17 Clyde Road, Bristol. |
| Embleton, Dennis C. ... ... ... ... ... ... | St. Wilfrid's, Bournemouth. |
| Evans, G. M. ... ... ... ... ... ... ... ... | Bridport. |
| Evans, J. Fenton, M.B., C.M. Ed. ... ... | Royal Infirmary, Bristol. |
| Evans, W. G. ... ... ... ... ... ... ... ... | Beckington. |
| *Ewens, John ... ... ... ... ... ... ... ... | 20 Cotham New Road, Bristol. |
| Fairbanks, W., M.D., C.M. Ed. ... ... ... | Wells. |
| Fasulo, Violetta ... ... ... ... ... ... ... | 16 Via St. Agostino, Florence. |
| *Fawcett, W. J., M.B., M.Ch., B.A. Dub. ... | 8 Sion Hill, Clifton. |
| *Fendick, R. George ... ... ... ... ... ... | 3 Claremont Place, Clifton. |
| Fernie, James ... ... ... ... ... ... ... ... | Stratton St. Margaret. |
| Fiddian, Alexander P., M.B. Lond. ... ... | 5 Newport Road, Cardiff. |
| Finch, Thomas, M.D. St. And. ... ... ... | Westville, St. Mary Church. |
| Fisher, Fred. B. ... ... ... ... ... ... ... | West Walk, Dorchester. |
| Fletcher, Henry Studd ... ... ... ... ... | Lydbrook. |
| Ford, Joseph ... ... ... ... ... ... ... ... | Wedmore. |
| Forty, D. H. ... ... ... ... ... ... ... ... | Wotton-under-Edge. |
| Fox, Arthur E. W., M.B., C.M. Ed. ... ... | 16 Gay Street, Bath. |
| *Fox, Bonville B., M.D., M.A. Oxon. ... ... | Brislington. |
| Fox, Charles Henry, M.D. St. And. ... ... | The Beeches, Brislington. |
| *Fox, E. Long, M.D. Oxon. ... ... ... ... | Church House, Clifton. |
| Fraser, D. A. ... ... ... ... ... ... ... ... | Arranmore House, Burnham, Somerset. |
| Fraser, Græme B. ... ... ... ... ... ... ... | 7 Lower Church Road, Weston-s.-Mare. |
| Freeman, Henry W. ... ... ... ... ... ... | 24 Circus, Bath. |
| Fry, J. Farrant ... ... ... ... ... ... ... | 203 St. Helen's Road, Swansea. |
| Fry, John B. ... ... ... ... ... ... ... ... | Swindon. |
| Fuller, J. ... ... ... ... ... ... ... ... ... | Long Ashton. |
| Furse, Edwin ... ... ... ... ... ... ... ... | South Molton. |
| *Fyffe, W. Johnstone, M.D. Dub.... ... ... | 2 Rodney Place, Clifton. |
| Gabb, J. Edward ... ... ... ... ... ... ... | 10 Oxford Place, Cheltenham. |
| Gamble, C. H. ... ... ... ... ... ... ... ... | Barnstaple. |
| Gardiner, George ... ... ... ... ... ... | 1 Redcliff Hill, Bristol. |
| *Gibbs, Alfred N. Godby ... ... ... ... ... | 27 Chandos Road, Bristol. |
| Gibbs, Robert ... ... ... ... ... ... ... | Orchard House, Teignmouth. |
| Gilbertson, Richard ... ... ... ... ... ... | Aberystwith. |
| Giles, G. F., M.D. ... ... ... ... ... ... | Lower Redland Road, Bristol. |
| *Gloag, G. A. ... ... ... ... ... ... ... ... | 6 College Green, Bristol. |
| Goodden, Wyndham C. ... ... ... ... ... | 86 Stapleton Road, Bristol. |
| Gooding, J. Callender, M.D. Ed. ... ... | Berkeley Street, Cheltenham. |
| Goodridge, Henry F. A., M.D. Lond. ... | 10 Brook Street, Bath. |

### LIST OF SUBSCRIBERS.

| | |
|---|---|
| Goss, T. Biddulph | 36 Paragon, Bath. |
| Gowing, B. C. | Avon House, Salisbury. |
| Grace, Alfred | Chipping Sodbury. |
| Grace, Edward Mills | Park House, Thornbury. |
| Grace, Henry | Kingswood, Bristol. |
| Grace, W. G. | 57 Stapleton Road, Bristol. |
| Gray, F. A. | Ottery St. Mary. |
| Green, F. K. | 3 Gay Street, Bath. |
| Gregory, George S. | Vernon Chambers, Southam'n Row, Lond. |
| *Griffiths, L. M. | 9 Gordon Road, Clifton. |
| Grote, G. W. | 32 Portland Square, Bristol. |
| Guinness, T. A. | Bampton, Devon. |
| *Hailes, Clement D. G., M.B., C.M. Ed. | 11 King's Parade, Clifton. |
| Harper, Joseph | Bear Street, Barnstaple. |
| *Harrison, A. J., M.B. Lond. | Guthrie Road, Clifton. |
| *Harsant, W. H. | 16 Pembroke Road, Clifton. |
| *Harwood, Septimus, M.B., C.M. Ed. | 3 Portland Square, Bristol. |
| Hawkins, Cæsar F. | 7 Berkeley Square, Bristol. |
| Haydon, Edgar, M.B., C.M. Glasg. | The Laurels, Newton Abbot. |
| Hayman, A. G. | 1 Pembroke Road, Clifton. |
| Heane, W. C. | Cinderford. |
| Hearder, G. J., M.D. St. And. | Joint Counties Asylum, Carmarthen. |
| *Heaven, John C. | Avonmouth. |
| *Herapath, C. K. C. | 11 Brunswick Square, Bristol. |
| *Hetling, H. E. | Stapleton Road, Bristol. |
| Hichens, James S. | 17 Green Lane, Redruth. |
| Hills, Frederic F. | 199 Cheltenham Road, Bristol. |
| Hinton, Joseph | Warminster. |
| Hitchman, John, M.D. St. And. | The Laurels, Fairford. |
| Hoblyn, Francis Parker | 10 Bladud Buildings, Bath. |
| Hooker, Charles P. | 104 Dyer Street, Cirencester. |
| Hopkins, H. Culliford | 4 Gay Street, Bath. |
| Hospital, Bristol General (*Sec.*, W. Thwaites). | |
| Howells, William, M.B., C.M. Glasg. | Church House, Talgarth. |
| Howse, William | 8 London Street, New Swindon. |
| Howsin, E. Arthur, M.D. St. And. | Stroud. |
| Hudson, H. R. | 182 Commercial Road, Newport, Mon. |
| Hunt, A. D. | Millbrook House, Chagford. |
| Hyatt, Brownlow N. | Park View, Shepton Mallet. |
| *Imlay, G. A., M.B., C.M. Aberd. | 4 Apsley Villas, Bristol. |
| Infirmary, Bristol Royal (*House Surgeon*, John Dacre.) | |
| Institution, Liverpool Medical (*Hon. Treas.* Dr. James Barr.) | |
| *Isaac, G. Washington, M.B., C.M. Ed. | 8 King's Parade, Clifton. |
| Jackson, J. B., M.D., M. Ch. Roy. Univ. I. | 56 Lemon Street, Truro. |
| Jago, Fred. W. P., M.B. Lond. | 16 Lockyer Street, Plymouth. |
| James, J. Bowen | Woodlands, Aberaman. |
| Jenkins, John Henry | Liskeard. |
| Jessop, Charles Moore | 98 Sutherland Gardens, London, W. |
| Jones, E. R. | Cilgynlle-fawr, New Quay, Cardiganshire |
| Jones, Evan | Ty-mawr, Aberdare. |
| Jones, W. R., M.B., C.M. Glasg. | Senny Bridge. |
| Joynes, Francis James | Eagle House, Dursley. |
| *Keall, W. P. | Coronation Road, Bristol. |
| Kempe, Arthur W. | 20 St. Sidwell's, Exeter. |

### LIST OF SUBSCRIBERS.

| | |
|---|---|
| KERR, ELIAS W., M.B., M.Ch., B.A. Dub. ... | Cerne Abbas. |
| KESTEVEN, W. B., M.D. St. And. ... ... ... | Little Park, Enfield. |
| KING, LOUIS J.... ... ... ... ... ... ... ... | 10 Russel Street, Bath. |
| KINNEIR, R. ... ... ... ... ... ... ... ... ... | The Tower House, Malmesbury. |
| KIRKMAN, J. MILLER ... ... ... ... ... ... ... | Wootton Bassett. |
| LAING, W. A. GORDON, M.B., C.M. Glasg. ... | Mevagissey. |
| LAMBE, J. ... ... ... ... ... ... ... ... ... ... | West Villa, Shurdington. |
| *LANSDOWN, F. POOLE ... ... ... ... ... ... | 19 White Ladies Road, Clifton. |
| *LAWRENCE, A. E. AUST, M.D., C.M. Aberd. | 15 Richmond Hill, Clifton. |
| *LAWRENCE, HENRY... ... ... ... ... ... ... | 9 Park Row, Bristol. |
| *LAXTON, HENRY ... ... ... ... ... ... ... ... | 12 Apsley Road, Clifton. |
| LEACH, J. COMYNS, M.D. Dur. ... ... ... ... | Sturminster Newton. |
| LEAMON, MICHAEL T. ... ... ... ... ... ... ... | 1 Bedford Villas, Tavistock. |
| *LEES, E. LEONARD, M.B., C.M. Ed. ... ... | Children's Hospital, Bristol. |
| LEWELLYN, JOHN ... ... ... ... ... ... ... ... | Caerphilly. |
| LIBRARY, BRISTOL MUSEUM AND ... ... ... | Queen's Road, Bristol. |
| LIBRARY, CHELTENHAM ... ... ... ... ... ... ... | 5 Royal Crescent, Cheltenham. |
| LIDDON, WILLIAM, M.B. Lond. ... ... ... | Church Square, Taunton. |
| LLOYD, WALTER E. ... ... ... ... ... ... ... | 60 York Road, Bristol. |
| LODGE, JOHN ... ... ... ... ... ... ... ... ... | Meridian House, Keynsham. |
| LOGAN, FREDERIC T. B. ... ... ... ... ... ... | Dean Lane, Bristol. |
| LOVELL, W. DAY ... ... ... ... ... ... ... ... | The Abbey, Bradford-on-Avon. |
| LOVELL, WILLIAM FREDERICK ... ... ... ... | West End House, Compton Martin. |
| LOWE, T. PAGAN ... ... ... ... ... ... ... ... | 1 Alfred Street, Bath. |
| *LUFFMAN, W. C. ... ... ... ... ... ... ... ... | 8 Cotham New Road, Bristol. |
| *LYONS, A. DE COURCY, M.B., C.M. Aberd. ... | Henley Lodge, Yatton. |
| *LYSAGHT, W. C. ... ... ... ... ... ... ... ... | 13 Frederick Place, Clifton. |
| MACDONALD, P. W., M.B., C.M. Aberd. ... | County Asylum, Dorchester. |
| MACLEAN, J. CAMPBELL, M.B., C.M. Ed. ... | Swindon House, Swindon. |
| MANNING, G. H., M.B., B. Ch., B.A. Dub. ... | Combe Martin. |
| MANNING, H. J. ... ... ... ... ... ... ... ... | Laverstock House, Salisbury. |
| MARLEY, HENRY F. ... ... ... ... ... ... ... | The Nook, Padstow. |
| MARSH, CHARLES J. ... ... ... ... ... ... ... | Yeovil. |
| MARSH, O. E. BULWER ... ... ... ... ... ... | 35 Bridge Street, Newport, Mon. |
| *MARSHALL, HENRY, M.D. Ed., F.R.S.E. ... | 28 Caledonia Place, Clifton. |
| *MARTIN, E. F., M.B. Ed. ... ... ... ... ... ... | Victoria House, Weston-super-Mare. |
| MARTIN, THEODORE ... ... ... ... ... ... ... | Temple Cloud. |
| MASON, FREDERICK ... ... ... ... ... ... ... | 20 Belmont, Bath. |
| MASON, S. B. ... ... ... ... ... ... ... ... ... ... | 12 Park Terrace, Pontypool. |
| MASON, WILLIAM ... ... ... ... ... ... ... ... | St. Austell. |
| *MATTHEWS, CHARLES E. ... ... ... ... ... ... | St. Paul's Road, Clifton. |
| *MAYOR, T. O. ... ... ... ... ... ... ... ... ... | 40 Apsley Road, Clifton. |
| MCNICOLL, JOHN ... ... ... ... ... ... ... ... | High Street, Poole. |
| *MEAD, H. RIMMINGTON ... ... ... ... ... ... | Fishponds. |
| MEREDITH, JOHN, M.D. Ed. ... ... ... ... | Wellington, Somersetshire. |
| METFORD, J. SEYMOUR ... ... ... ... ... ... ... | 31 Berkeley Square, Bristol. |
| MICHELL, GEORGE A. ... ... ... ... ... ... ... | Redruth. |
| MICHELL, SLOANE ... ... ... ... ... ... ... ... | Dolton. |
| MILNE, JOHN, M.B., C.M. Aberd. ... ... ... | 97 Clarence Road, Bristol. |
| MILWARD, JAMES ... ... ... ... ... ... ... ... | 54 Charles Street, Cardiff. |
| MORGAN, JOHN ... ... ... ... ... ... ... ... ... | Langport. |
| MORGAN, WILLIAM, M.D. Aberd. ... ... ... | 16 Edwards Terrace, Cardiff. |
| MORRIS, J. A. ... ... ... ... ... ... ... ... ... | Caerleon. |
| MOYNAN, W. ARTHUR, M.D., M.Ch. Qu. Univ. I. | St. Ann's Heath, Chertsey. |

### LIST OF SUBSCRIBERS.

| | |
|---|---|
| MUDGE, T.... ... ... ... ... ... ... ... ... | Towerhill, Bodmin. |
| MUNDEN, CHARLES ... ... ... ... ... ... ... | Ilminster. |
| MURRAY, WILLIAM, M.B., C.M. Ed. ... ... | Staple Hill. |
| NAPIER, T. W. A., M.B., C.M. Aberd. ... ... | Strathwye, Tintern. |
| *NEWMAN, CHARLES ... ... ... ... ... ... | 19 Portland Square, Bristol. |
| *NEWNHAM, W. H. C., M.B., M.A. Cantab. ... | General Hospital, Bristol. |
| NEWSTEAD, JAMES ... ... ... ... ... ... ... | 9 York Place, Clifton. |
| NEWTON, CHARLES JOHN ... ... ... ... ... | Oriel Lodge, Cheltenham. |
| NICHOLSON, T. D., M.D., C.M. Ed. ... ... | 2 White Ladies Road, Clifton. |
| NORMAN, GEORGE ... ... ... ... ... ... ... | 12 Brock Street, Bath. |
| NORMAN, J. H. ... ... ... ... ... ... ... ... | Goodleigh, Winkleigh. |
| NORMAN, J. W.... ... ... ... ... ... ... ... | Edde Cross House, Ross. |
| *NORTON, J. A., M.D., C.M. Aberd. ... ... ... | 8 Redcliff Hill, Bristol. |
| *NORTON, T. CHALMERS ... ... ... ... ... | 12 King Square, Bristol. |
| NOURSE, W. J. CHICHELE ... ... ... ... ... | Morchard Bishop. |
| OLLERHEAD, T. J. ... ... ... ... ... ... ... | Hillbury, Minehead. |
| *ORMEROD, HENRY ... ... ... ... ... ... ... | Westbury-upon-Trym. |
| PADLEY, GEORGE ... ... ... ... ... ... ... | 2 Northampton Terrace, Swansea. |
| PAINE, WILLIAM HENRY, M.D. Aberd. ... ... | Stroud. |
| PALM, THEOBALD A., M.D., C.M., M.A. Ed.... | Greenhill, Thorncombe. |
| PALMER, F. CRADDOCK ... ... ... ... ... ... | The Corderries, Chalford. |
| PARRY, J. H. ... ... ... ... ... ... ... ... | 99 Ashley Road, Bristol. |
| PARSLOE, H. H. ... ... ... ... ... ... ... | The Larches, Black Torrington. |
| *PARSON, T. COOKE ... ... ... ... ... ... ... | 21 White Ladies Road, Bristol. |
| PARSONS, FRANCIS J. C. ... ... ... ... ... | King Square, Bridgwater. |
| PARSONS, FRED. ... ... ... ... ... ... ... | Willow Vale, Frome. |
| PARSONS, J. D. F., M.D. St. And. ... ... ... | 13 Dowry Square, Clifton. |
| PAULI, G. C. P. ... ... ... ... ... ... ... | 240 York Road, Bristol. |
| PAULL, JOSIAH ... ... ... ... ... ... ... ... | Camborne. |
| PEARCE, EDWARD ... ... ... ... ... ... ... | Camelford. |
| PEARSE, FRED. E., M.D. St. And. ... ... ... | Hall House, Frome. |
| *PENNY, W. J. ... ... ... ... ... ... ... ... | 42 Caledonia Place, Clifton. |
| PENRUDDOCKE, CHARLES ... ... ... ... ... | Winchcombe. |
| PENTLAND, YOUNG J. ... ... ... ... ... ... | 11 Teviot Place, Edinburgh. |
| PERKINS, J. H., M.D. New York ... ... ... | 14 Victoria Square, Clifton. |
| PESKETT, A. W. C., M.B., B.C., M.A. Cantab. | Burnham, Somersetshire. |
| PHELPS, W. HARFORD G., M.D. Aberd. ... | Beachfield, Weston-super-Mare. |
| PHELPS, WILLIAM H. ... ... ... ... ... ... | 123 Ashley Road, Bristol. |
| PHILLIPS, E. ENGLAND ... ... ... ... ... ... | Broadwater House, Southend. |
| PHILLIPS, J. W. ... ... ... ... ... ... ... ... | Cowbridge. |
| PHILPOTS, EDWARD P., M.D., C.M. Aberd. ... | Bourne Hall, Bournemouth. |
| PHILPOTS, JOHN R. ... ... ... ... ... ... | Parkstone. |
| *PICKERING, C. F. ... ... ... ... ... ... ... | 12 Berkeley Square, Bristol. |
| PIPPETTE, WALTER... ... ... ... ... ... ... | Wilton, Wiltshire. |
| PIZEY, G. F. P.... ... ... ... ... ... ... ... | Rydal Villa, Clevedon. |
| *POUNTNEY, W. E., M.D., C.M. Ed. ... ... | 107 White Ladies Road, Bristol. |
| POWELL, WILLIAM, M.B. Lond. ... ... ... | Hill Garden, Torquay. |
| PRICE, WILLIAM, M.B. Lond. ... ... ... ... | 3 Park Place, Cardiff. |
| *PRICHARD, ARTHUR W.... ... ... ... ... ... | Gordon Road, Clifton. |
| *PRICHARD, AUGUSTIN, M.D. Berlin ... ... ... | 4 Chesterfield Place, Clifton. |
| *PRICHARD, J. E., M.B., B.A. Oxon. ... ... | 243 Cheltenham Road, Bristol. |
| PRIDEAUX, T. ENGLEDUE P. ... ... ... ... | Wellington, Somersetshire. |
| *PRING, ARTHUR, B.A. Cantab. ... ... ... ... | 4 Pembroke Road, Clifton. |
| *PROWSE, ARTHUR B., M.D. Lond. ... ... ... | 4 Rodney Cottages, Clifton. |

### LIST OF SUBSCRIBERS.

| | |
|---|---|
| Pullin, Bingley G. | Sidmouth. |
| Pullin, Thomas H. S., M.D. St. And. | Sidmouth. |
| Randolph, Charles | St. Michael's, Milverton, Somerset. |
| Rawlings, John Adams | 4 Northampton Terrace, Swansea. |
| Reader, Jeremiah | Marshfield. |
| Redwood, T. Hall, M.D., C.M., M.A. Dur. | The Lawn, Rhymney. |
| Rees, D. Valentine | Lion Street, Brecon. |
| Rendall, John | Lymington. |
| Riddell, Robert, M.D., M.Ch. Qu. Univ. I. | East Hill Lodge, Dunsford. |
| Robothan, G. B. | The Grove, Risca. |
| Rogers, George, M.D. St. And. | 6 Portland Square, Bristol. |
| Rolston, G. T. | 8 Osborne Villas, Stoke, Devonport. |
| Rolston, John R. | 5 St. Aubyn Street, Devonport. |
| Rossiter, George F., M.B. Lond. | Cairo Lodge, Weston-super-Mare. |
| *Roué, W. Barrett, M.D., M.S. Dur. | 2 Buckingham Place, Clifton. |
| Row, Charles | Lostwithiel. |
| Row, William | Lerrin, Lostwithiel. |
| Rowe, Edmund L. | County Asylum, Gloucester. |
| *Roxburgh, Robert, M.B. Ed. | 1 Victoria Buildings, Weston-super-Mare. |
| *Rudge, C. King | 145 White Ladies Road, Bristol. |
| *Rudge, H. T. | 5 Colston's Parade, Bristol. |
| Rundle, Edmund | Royal Cornwall Infirmary, Truro. |
| Rundle, Henry | 11 Clarence Parade, Southsea, Hampshire. |
| Runnalls, H. Boyle | 118 Fore Street, Saltash. |
| Russell, M. W. H. | Royal United Hospital, Bath. |
| Salmon, W. G. | Thornbury. |
| Sanctuary, Thomas, M.D. Ed. | 29 Castle Street, Salisbury. |
| Scott, James, M.B., C.M. Ed. | H.M. Prison, Hyde Road, Manchester. |
| Scott, Richard J. H. | 13 Bladud Buildings, Bath. |
| Scott, W. G., M.B., C.M. Ed. | Newton Abbot. |
| Searle, George C. | Brixham. |
| Searle, Richard Burford | St. Just. |
| Seward, W. J., M.B. Lond. | County Asylum, Colney Hatch, Lond., N. |
| Sewart, J. H. | Lostwithiel. |
| Shapter, Lewis, M.D. Cantab. | The Barnfield, Exeter. |
| *Shaw, J. E., M.B., C.M. Ed. | 11 Lansdown Place, Clifton. |
| Sheehy, J. | Victoria, Ebbw Vale. |
| Sheen, A., M.D. St. And. | 23 Newport Road, Cardiff. |
| Sheldon, T. Steele, M.B. Lond. | Parkside House, Macclesfield. |
| *Sheppard, E. J. | 16 Park Place, Clifton. |
| *Shorland, Walter E. | 189 Cheltenham Road, Bristol. |
| Shortridge, Thomas Wood, M.D. Brussels | Honiton. |
| Sincock, J. Bain | Friarn Street, Bridgwater. |
| Skeate, Edwin | 16 Paragon, Bath. |
| *Skelton, Henry, M.D. St. And. | Downend. |
| *Skerritt, E. Markham, M.D., B.S., B.A. Lond. | Richmond Hill, Clifton. |
| *Skinner, Stephen, M.B., C.M. Aberd. | Ferndale, Clevedon. |
| *Smith, George | The Coombe, Westbury-upon-Trym. |
| *Smith, G. Munro | 70 Pembroke Road, Clifton. |
| *Smith, J. Greig, M.B., C.M., M.A. Ab., F.R.S.E. | 16 Victoria Square, Clifton. |
| Smith, J. Sidney, M.D. St. And. | Tiverton. |
| *Smith, R. Shingleton, M.D., B.Sc. Lond. | 9 Richmond Hill, Clifton. |
| Smith, Samuel | 7 Kingsdown Parade, Bristol. |

Society, Cardiff Medical (*Hon. Sec.*, T. Garrett Horder).

### LIST OF SUBSCRIBERS.

| | |
|---|---|
| SOCIETY, PLYMOUTH MEDICAL (*Hon. Sec.*, C. WHIPPLE). | |
| SOCIETY, ROYAL MEDICAL AND CHIRURGICAL | 53 Berners Street, London, W. |
| SOMER, JAMES ... ... ... ... ... ... ... ... ... | Broadclyst. |
| *SPENCER, W. H., M.D., M.A. Cantab. ... ... | 5 Lansdown Place, Clifton. |
| SPENDER, JOHN K., M.D. Lond. ... ... ... | 17 Circus, Bath. |
| STANSFELD, G. M. ... ... ... ... ... ... ... ... | 19 Victoria Square, Clifton. |
| STATHAM, R. WHITESIDE ... ... ... ... ... ... | Cheddar. |
| STEEL, ARTHUR R.... ... ... ... ... ... ... ... | Lockridge Cottage, Beer Alston. |
| STEEL, S. H., M.B. Lond. ... ... ... ... ... | Abergavenny. |
| STEEL, WILLIAM D., M.D., C.M. Aberd. ... | Abergavenny. |
| *STEELE, CHARLES, M.D. Dur. ... ... ... ... | 17 Richmond Hill, Clifton. |
| STEELE-PERKINS, E. B. ... ... ... ... ... ... | 1 Hill's Buildings, St. Sidwell's, Exeter. |
| *STEWART, JAMES, B.A. Qu. Univ. I. ... ... | Dunmurry, Stoke Bishop. |
| STOCK, G. ... ... ... ... ... ... ... ... ... ... | Barton Hill Road, Bristol. |
| STOCKWELL, FRED., M.D. Lond. ... ... ... | Bruton. |
| STODDART, F. W. ... ... ... ... ... ... ... ... | 1 Park Street, Bristol. |
| STORRY, FREDERICK W. ... ... ... ... ... ... | Lansdown, Stroud. |
| *SWAYNE, J. G., M.D. Lond. ... ... ... ... | 74 Pembroke Road, Clifton. |
| *SWAYNE, S. H.... ... ... ... ... ... ... ... ... | 129 Pembroke Road, Clifton. |
| SYDENHAM, GEORGE F. ... ... ... ... ... | Brushford, Exbridge. |
| SYDNEY-TURNER, A. M. ... ... ... ... ... ... | College Green, Gloucester. |
| SYMES, JAMES ... ... ... ... ... ... ... ... ... | Briton Ferry. |
| TAYLER, GEO. CHRISTOPHER, M.D. Lond. ... | Trowbridge. |
| *TAYLOR, J. ... ... ... ... ... ... ... ... ... ... | 5 Ashley Road, Bristol. |
| TAYLOR, THOMAS HENRY ... ... ... ... ... | Prospect House, Thornbury. |
| TERRY, GEORGE ... ... ... ... ... ... ... ... | Mells. |
| THOMAS, A. GARROD, M.D., C.M. Ed. ... ... | 28 Bridge Street, Newport, Mon. |
| THOMAS, JABEZ ... ... ... ... ... ... ... ... | Ty Cerrig, Swansea. |
| THOMAS, R. W. ... ... ... ... ... ... ... ... | Tregare House, Keynsham. |
| *THOMPSON, GEORGE, M.D. Dur. ... ... ... | City and County Asylum, Stapleton. |
| THOMPSON, JOHN, M.D., St. And. ... ... ... | Lynton House, Bideford. |
| THOMSON, G. S., M.D. Ed. ... ... ... ... | 8 College Road, Clifton. |
| THOMSON, SPENCER, M.D. St. And. ... ... | Ashton, Torquay. |
| *THURSTON, HUGH C. ... ... ... ... ... ... | Royal Infirmary, Bristol. |
| *TIVY, W. J. ... ... ... ... ... ... ... ... ... | 8 Lansdown Place, Clifton. |
| TODD, W. J. ... ... ... ... ... ... ... ... ... | North Petherton. |
| TOMKINS, HARDING H.... ... ... ... ... ... ... | County Asylum, Gloucester. |
| TORBOCK, W. H. ... ... ... ... ... ... ... ... | Polruan. |
| TOSSWILL, LOUIS H., M.B. Cantab. ... ... | 34 West Southernhay, Exeter. |
| TROTTER, LESLIE B., M.D., C.M. Ed. ... ... | Coleford, Gloucestershire. |
| TWINING, A. H. ... ... ... ... ... ... ... ... | The Knoll, Salcombe. |
| TYLEY, R. P., M.D. St. And. ... ... ... ... | Wedmore. |
| URWICK, W. ... ... ... ... ... ... ... ... ... | New Quay, Cornwall. |
| VACHELL, C. T., M.D. Lond. ... ... ... ... | 38 Charles Street, Cardiff. |
| VACHELL, HERBERT R., M.D., C.M. Aberd.... | 45 Crockherbtown, Cardiff. |
| WADE, A. LAW, M.D. Dub. ... ... ... ... | Somerset County Asylum, Wells. |
| *WALDO, HENRY, M.D., C.M. Aberd. ... ... | 19 Pembroke Road, Clifton. |
| WALLACE, REV. C. HILL, M.A. Oxon. ... ... | 3 Harley Place, Clifton. |
| WALSH, DAVID H. ... ... ... ... ... ... ... | Ivywell House, Stoke Bishop. |
| WALTER, WILLIAM HENRY ... ... ... ... ... | Knapp Villa, South Petherton. |
| WARD, J. L. W. ... ... ... ... ... ... ... ... | Victoria Street, Merthyr Tydvil. |
| *WARNER, E. H., M.D., C.M. Ed. ... ... ... | Barton Hill House, Bristol. |
| *WATHEN, J. HANCOCKE ... ... ... ... ... | 16 York Place, Clifton. |
| WATSON, J. ADAM ... ... ... ... ... ... ... | Chudleigh. |

| | |
|---|---|
| WATTERS, GEORGE T. B., M.D., C.M. Ed. ... | Stonehouse. |
| WATTS, THOMAS ... ... ... ... ... ... ... | Frampton-on-Severn. |
| WAUGH, ALEXANDER ... ... ... ... ... ... | Midsomer Norton. |
| *WEATHERLY, LIONEL A., M.D., C.M. Aberd. | Portishead. |
| WEBB, W. H. ... ... ... ... ... ... ... ... | Kingsbridge. |
| *WEBSTER, THOMAS ... ... ... ... ... ... | Redland Hill, Bristol. |
| WEIR, FREDERICK W. ... ... ... ... ... ... | 1 Dowry Parade, Bristol. |
| *WELSH, F. F. ... ... ... ... ... ... ... ... | Rockleaze Point, Stoke Bishop. |
| WHITELEY, GEORGE W. ... ... ... ... ... | Downton, Wiltshire. |
| WHITFELD, W. CLARKE ... ... ... ... ... | 5 St. Ethelbert Street, Hereford. |
| WICKHAM, W. ... ... ... ... ... ... ... ... | Hill House, Tetbury. |
| WICKSTEED, FRANCIS W. S. ... ... ... ... | Chester House, Weston-super-Mare. |
| WIGMORE, J., M.D. Qu. Univ. I.... ... ... ... | Twerton-on-Avon. |
| WILLCOX, R. LEWIS ... ... ... ... ... ... | Warminster. |
| WILLETT, M., M.D. St. And. ... ... ... ... | 87 Ashley Road, Bristol. |
| WILLIAMS, CHARLES ... ... ... ... ... ... | Port Isaac. |
| WILLIAMS, D. J. ... ... ... ... ... ... ... | Greenfield House, Llanelly. |
| WILLIAMS, H. E. ... ... ... ... ... ... ... | The Mount, Newport, Mon. |
| WILLIAMS, PETER ... ... ... ... ... ... ... | Beach House, Ferryside. |
| WILLIAMS, WILLIAM HENRY, M.D. Lond. ... | Sherborne. |
| WILSON, GEORGE ... ... ... ... ... ... ... | Chippenham House, Monmouth. |
| WILTON, JOHN P. ... ... ... ... ... ... ... | 10 College Green, Gloucester. |
| WINTERBOTHAM, L. ... ... ... ... ... ... | Bays Hill, Cheltenham. |
| WISE, N. V. ... ... ... ... ... ... ... ... | Fore Street, Trowbridge. |
| WOODD, H. T. ... ... ... ... ... ... ... ... | Calstock. |
| WORKMAN, C. J., M.D. Ed. ... ... ... ... | Teignmouth. |
| YELF, LEONARD K., M.D. St. And. ... ... ... | Moreton-in-Marsh. |

### Exchange-List.

### AMERICA.

### ARGENTINE REPUBLIC.

*Monthly.*

*Anales del Circulo Médico Argentino.* Buenos Ayres: M. Biedma.

### CANADA.

*Monthly.*

*Canada Medical and Surgical Journal.* Montreal: Gazette Printing Company.

### PERU.

*Monthly.*

*La Crónica Médica.* Lima: Carlos Prince.

### UNITED STATES.

*Weekly.*

*The Medical and Surgical Reporter.* Philadelphia: 115 South Seventh Street.

*The Medical Record.* New York: William Wood & Co.

*The New York Medical Journal.* New York: D. Appleton & Co.

*The Cincinnati Lancet-Clinic.* Cincinnati: 281 West 7th Street.

*The Journal of the American Medical Association.* Chicago: 65 Randolph Street.

*Fortnightly.*

*Philadelphia Medical Times.* Philadelphia: J. B. Lippincott & Co.

*The American Practitioner & News.* Louisville: John P. Morton and Company.

*Monthly.*

*Index Medicus.* Detroit: G. S. Davis.

*Journal of Cutaneous and Venereal Diseases.* New York: William Wood & Company.

*Pacific Medical and Surgical Journal and Western Lancet.* San Francisco: W. S. Duncombe.

*The American Journal of Ophthalmology.* St. Louis: J. H. Chambers & Co.

*The Medical Analectic.* New York: G. P. Putnam's Sons.

*The Obstetric Gazette.* Cincinnati: 281 West 7th Street.

*The Polyclinic.* Philadelphia: P. Blakiston, Son & Co.

*The Saint Louis Medical and Surgical Journal.* Saint Louis: 2622 Washington Avenue.

*The Therapeutic Gazette.* Detroit: George S. Davis.

*Quarterly.*

*The American Journal of the Medical Sciences.* Philadelphia: Lea Brothers & Co.

*Yearly.*

*Transactions of the American Surgical Association.* Philadelphia: P. Blakiston, Son & Co.

*Occasionally.*

*Transactions of the New York Academy of Medicine.* New York: 12 West 31st Street.

### ASIA.

### INDIA.

*Monthly.*

*The Indian Medical Journal.* Calcutta: W. Newman & Co., Ld.

### JAPAN.

*Monthly.*

*Transactions of the Sei-I-Kwai.* Tokio: Z. P. Maruya & Co.

### AUSTRALASIA.

### AUSTRALIA.

*Monthly.*

*The Australasian Medical Gazette.* Sydney: L. Bruck.

*The Australian Medical Journal.* Melbourne: Stillwell & Co.

### EUROPE.

### BELGIUM.

*Monthly.*

*Bulletin de l'Académie royale de médecine de Belgique.* Brussels: A. Manceaux.

### BRITISH ISLES.

*Weekly.*

*British Medical Journal.* London: 161 A Strand.

*The Lancet.* London: 423 Strand.

*Monthly.*

*Annals of Surgery.* London: Baillière, Tindall & Cox.

*The Birmingham Medical Review.* Birmingham: Cornish Bros.

*The London Medical Record.* London: Smith, Elder, & Co.

*The Medical Chronicle.* Manchester: John Heywood.

*The Practitioner.* London: Macmillan & Co.

*Edinburgh Medical Journal.* Edinburgh: Oliver and Boyd.

*The Glasgow Medical Journal.* Glasgow: Alex. Macdougall.

*The Dublin Journal of Medical Science.* Dublin: Fannin & Co.

*Quarterly.*

*The Asclepiad.* London: Longmans, Green, & Co.

*The British Gynæcological Journal.* London: Smith, Elder, & Co.

*Half-yearly.*

*The Liverpool Medico-Chirurgical Journal.* Liverpool: Medical Institution.

*The Retrospect of Medicine.* London: Simpkin, Marshall, & Co.

*Yearly.*

*The Medical Annual.* London: Henry Kimpton.

*The Year-Book of Treatment.* London: Cassell & Company, Limited.

*Transactions of the Academy of Medicine in Ireland.* Dublin: Fannin and Company.

### DENMARK.

*Weekly.*

*Hospitals-Tidende.* Copenhagen: Jacob Lund.

### FRANCE.

*Three times a week.*

*Gazette des hôpitaux.* Paris: 4 Rue de l'Odéon.

*L'Union médicale.* Paris: 11 Rue de la Grange-Batelière.

*Weekly.*

*Bulletin de l'Académie de médecine.* Paris: G. Masson.

*Gazette médicale de Paris.* Paris: 8 Place de l'Odéon.

*Le Progrès médical.* Paris: 14 Rue des Carmes.

*Monthly.*

*Gazette de Gynécologie.* Paris: O. Doin.

*Revue mensuelle de Laryngologie, d'Otologie et de Rhinologie.* Paris: 8 Place de l'Odéon.

### GERMANY.

*Twice a week.*

*Deutsche Medizinal-Zeitung.* Berlin: Eugen Grosser.

*Weekly.*

*Medicinische Bibliographie.* Leipzig: Breitkopf & Härtel.

### GREECE.

*Weekly.*

*Γαληνὸς.* Athens: N. G. Passare.

### HOLLAND.

*Weekly.*

*Nederlandsch Tijdschrift voor Geneeskunde.* Amsterdam: F. van Rossen.

### ITALY.

*Monthly.*

*Lo Sperimentale.* Florence: 35 Via degli Alfani

### NORWAY.

*Monthly.*

*Norsk Magazin for Lægevidenskaben.* Christiania: Th. Steens.

### PORTUGAL.

*Weekly.*

*A Medicina Contemporanea.* Lisbon: José Antonio Rodrigues & Cª

### RUSSIA.

*Weekly.*

*Vrach.* St. Petersburg: C. Ricker

### SPAIN.

*Weekly.*

*El Siglo Médico.* Madrid: Magdalena, 36, 2º

### SWITZERLAND.

*Monthly.*

*Illustrirte Monatsschrift der ärztlichen Polytechnik und Centralblatt der orthopädischen Chirurgie.* Berne: Schmid, Francke & Co.

### Bristol Medico-Chirurgical Journal.

MARCH, 1886.

### CONTENTS.

Page.

ORIGINAL PAPERS:—

Removal of the Uterine Appendages. By J. GREIG SMITH ... ... 1

Relation between Facial Neuralgia and Dental Irritation. By J. M. ACKLAND, M.R.C.S., L.D.S. Eng. ... ... ... ... ... ... ... 28

CLINICAL RECORDS:—

A Case of Dislocation of the Ulna Backwards and the Radius Forwards. By R. J. H. SCOTT, M.R.C.S. Eng. ... ... ... ... 36

Calculus on Foreign Body in Bladder; Perforation of Bladder. No Symptoms. By H. BENHAM, M.D., and J. GREIG SMITH ... 38

Tonic Contraction of the Lower Extremities, or Tetany. By W. H. WEBB, M.R.C.S., L.S.A. ... ... ... ... ... ... ... ... 42

PERISCOPE OF MEDICAL AND SURGICAL PROGRESS:—

Notes on Medical Therapeutics. By ARTHUR B. PROWSE, M.D. Lond., F.R.C.S. Eng. ... ... ... ... ... ... ... ... ... ... ... ... 45

Excoriationes Narium. By BARCLAY J. BARON, M.B. ... ... ... 52

Some Recent Additions to the Dietary of the Sick. By R. SHINGLETON SMITH, M.D. Lond., F.R.C.P. ... ... ... ... ... ... ... ... 55

REVIEWS AND NOTICES OF BOOKS ... ... ... ... ... ... ... ... ... 60

SCRAPS ... ... ... ... ... ... ... ... ... ... ... ... ... ... ... 71

---

*Cases for binding, price 7d. each, may be had of the Publisher,*

J. W. ARROWSMITH, *Quay Street, Bristol.*

### *BOOKS AND PAMPHLETS RECEIVED.*

From H. K. Lewis, London:
*Lewis' Pocket Medical Vocabulary.*
*On the Fœtus in Utero.* By Alexander Harvey, M.D.
*Rome in Winter, and the Tuscan Hills in Summer.* By David Young, M.D.
*Materia Medica and Pharmacy.* By Alfred W. Gerrard.
*Diseases of the Lungs.* Third Edition. By R. Douglas Powell, M.D.
*A Guide to the Examination of the Nose.* By E. Cresswell Baber, M.B.
*Elements of Practical Medicine.* By Alfred H. Carter, M.D.
*A Guide to the New Pharmacopœia.* Second Edition.

From J. & A. Churchill, London:
*How to use a Galvanic Battery.* Third Edition. By Herbert Tibbits.
*Hunterian Lectures.* By Edward Lund, F.R.C.S.
*Outlines of Infectious Diseases.* By James W. Allan, M.B.
*The Prescriber's Pharmacopœia.* Sixth Edition. By Nestor Tirard, M.D.
*Handbook of the Diseases of the Nervous System.* By James Ross, M.D., LL.D.
*On the Supra-pubic operation of opening the Bladder.* By Sir Henry Thompson.

From Smith, Elder & Co., London:
*A Guide to Therapeutics.* Fourth Edition. By R. Farquharson, M.D., M.P.

From Cassell & Co., London:
*The Surgical Diseases of Children.* By Edmund Owen, M.B. 1885.
*Comparative Anatomy and Physiology.* By F. Jeffrey Bell, M.A. 1885.
*Fractures and Dislocations.* By T. Pickering Pick, F.R.C.S. 1885.
*Surgical Diseases of the Kidney.* By Henry Morris, M.A., M.B. 1885.

From Young J. Pentland, Edinburgh:
*Atlas of Venereal Diseases.* Fasc. II., III., IV. By P. H. Maclaren.

From P. Blakiston & Co., Philadelphia:
*The Field and Limitation of the Operative Surgery of the Human Brain.* By John B. Roberts, A.M., M.D.

From Henry Renshaw, London:
*Five Hundred Cases of Fistula, Piles, &c.* By Samuel Benton, M.R.C.S.

From David Torres Aguirre, Lima:
*El Monitor Medico.* No. 11.

From Bigelow Brothers, Buffalo, N.Y.:
*The Medical Press.* Vol. I. Nos. 3, 4.

From Trow's Printing and Bookbinding Company, New York:
*Diphtheria and its Management.* By Joseph E. Writings, M.D. 1885. Reprint.

From Stefan Mihalescu, Bucuresci:
*Spitalul.* Anul. VI. No. 1.

### Bristol Medico-Chirurgical Society.

---

**President.**

W. H. SPENCER, M.D., M.A. Cantab.

**President-Elect.**

F. POOLE LANSDOWN.

**Honorary Secretary.**

R. SHINGLETON SMITH, M.D., B.Sc. Lond

**Committee.**

*Past Presidents.*
R. W. COE.
AUGUSTIN PRICHARD, M.D. Berlin.
W. MICHELL CLARKE.
J. G. SWAYNE, M.D. Lond.
E. LONG FOX, M.D. Oxon.
JAMES G. DAVEY, M.D. St. And.
W. JOHNSTONE FYFFE, M.D. Dub.
G. F. BURDER, M.D., Aberd.

F. RICHARDSON CROSS, M.B. Lond.
NELSON C. DOBSON.
L. M. GRIFFITHS.
A. J. HARRISON, M.B. Lond.
E. MARKHAM SKERRITT, M.D., B.S., B.A. Lond.
J. GREIG SMITH, M.B., C.M., M.A. Aberd., F.R.S.E.

Medical Men wishing to join the Society are requested to communicate with the Honorary Secretary, Dr. R. SHINGLETON SMITH, 9 Richmond Hill, Clifton. The Annual Subscription is 10/6.

---

*Extracts from Journal By-laws.*

4.—The *Journal* shall be delivered free to Subscribers and also to Members of the Society whose subscriptions are not in arrear.

6.—The Annual Subscription to the *Journal* for those who pay before the 1st of July shall be 5/-, post free, or 6/- if paid after that date.

---

Communications intended for insertion in the *Journal*, Books for Review, &c., should be sent to the Editor, J. GREIG SMITH, M.A., F.R.S.E., 16 Victoria Square, Clifton.

Exchange Journals and communications referring to the delivery of the *Journal* should be sent to the Assistant-Editor, L. M. GRIFFITHS, 9 Gordon Road, Clifton, to whom Subscriptions are to be paid *in advance*.

Authors requiring Reprints of their Papers should communicate with the Publisher.

Cases for Binding Vols. I., II., and III. are now ready, and may be obtained, price 7*d*. each (postage 2*d*. extra), upon application to J. W. ARROWSMITH, Quay Street, Bristol; or the *Journal* will be bound, including Case, for 1/3 per volume.

